# Increased risk of overactive bladder in patients with idiopathic Parkinson’s disease: Insight from a nationwide population-based cohort study

**DOI:** 10.1371/journal.pone.0193783

**Published:** 2018-03-02

**Authors:** Fu-Yu Lin, Yi-Chien Yang, Cheng-Li Lin, Lukas Jyuhn-Hsiarn Lee

**Affiliations:** 1 Department of Neurology, China Medical University Hospital, China Medical University School of Medicine, Taichung, Taiwan; 2 College of Medicine, China Medical University, Taichung, Taiwan; 3 Management Office for Health Data, China Medical University Hospital, Taichung, Taiwan; 4 National Institute of Environmental Health Sciences, National Health Research Institutes, Miaoli, Taiwan; 5 Institute of Occupational Medicine and Industrial Hygiene, College of Public Health, National Taiwan University, Taipei, Taiwan; 6 Ph.D. Program in Environmental and Occupational Medicine, Kaohsiung Medical University, Kaohsiung, Taiwan; Hokkaido Daigaku, JAPAN

## Abstract

**Background:**

Idiopathic Parkinson’s disease (IPD) is a progressive neurodegenerative disorder characterized by typical motor impairment. However, lower urinary tract symptoms, including urinary urgency or frequency, which are non-motor phenomena, occur frequently among patients with IPD. In this study, we assess the risk of overactive bladder (OAB) in patients with IPD.

**Methods:**

The National Health Insurance Research Database of Taiwan was used to identify patients with IPD (IPD cohort) and four-fold controls (non-IPD cohort) from 2000 to 2010. The non-IPD cohort was matched according to age, sex, and baseline comorbidities, including benign prostate hyperplasia, stress incontinence, diabetes, and cerebrovascular diseases. The occurrence of OAB was monitored until the end of 2011. Hazard ratios of OAB were estimated using Cox proportional hazards regression models.

**Results:**

In total, 4,571 and 18,255 patients were included in IPD and non-IPD cohorts, respectively. Results showed a significantly higher overall incidence rate of OAB in the IPD cohort compared with the non-IPD cohort (14.5 vs. 6.37 per 10,000 person-years), with a 2.3-fold increased risk of OAB (95% confidence interval [CI] = 1.51–3.51) after controlling for benign prostate hyperplasia and stress incontinence. The mean follow-up period for the IPD cohort was 5.0 years. This cohort study showed that the cumulative incidence of OAB was 0.65% at the fifth year and 1.54% at the tenth year after IPD diagnosis; this risk was highest in the age group 65–74 years.

**Conclusion:**

This study reveals that IPD is independently associated with an increased risk of OAB in patients with IPD. The probability of OAB was 1.54% over a 10-year period after IPD diagnosis; the risk of OAB is considered to be age-dependent and most substantial in patients aged 65–74 years.

## Introduction

Idiopathic Parkinson’s disease (IPD) is a progressive neurodegenerative disorder characterized by early programmed cell death of dopaminergic neurons in the substantia nigra pars compacta [1]. It leads to movement impairment manifested as rest tremor, muscle rigidity, bradykinesia, postural imbalance, and gait disturbance [2]. The non-motor phenomena of IPD include neuropsychiatric disorders, sleep abnormalities, sensory symptoms, and autonomic impairments [3]. Among autonomic impairments, lower urinary tract symptoms (LUTS) are the most common manifestations [4,5]. Patients with IPD often experience urinary urgency or frequency, and urodynamic studies confirm the high prevalence of detrusor overactivity [6]. Although IPD may be pathophysiologically related to overactive bladder (OAB), as indicated by the morphological alteration of the brainstem raphe [7], a limited number of longitudinal epidemiological studies have documented the risk of OAB among patients with IPD [8]. To the best of our knowledge, there have been no large-scale studies reporting the possibility of OAB in patients of Asian descent; therefore, this paper presents a nationwide retrospective cohort study evaluating the risk of OAB in Chinese patients with IPD.

## Methods

### Data source

This retrospective cohort study used information pertaining to the year 2000 obtained from the Longitudinal Health Insurance Database (LHID). The LHID is a subset of the National Health Insurance Research Database (NHIRD), which comprises data claims from patients enrolled in the National Health Insurance Program (NHIP) and covers more than 99% of the 23 million residents in Taiwan [9]. The LHID comprises one million beneficiaries randomly selected from the NHIP registry in 2000 for research purposes. Details of the LHID have been provided in previous publications [10,11]. Clinical diagnoses for patients in the LHID were made according to the International Classification of Diseases, Ninth Revision, Clinical Modification (ICD-9-CM). This study involved a secondary analysis of health-related databases and was approved by the Ethics Review Board of China Medical University (CMUH-104-REC2-115).

### Study participants

**Fig 1:**
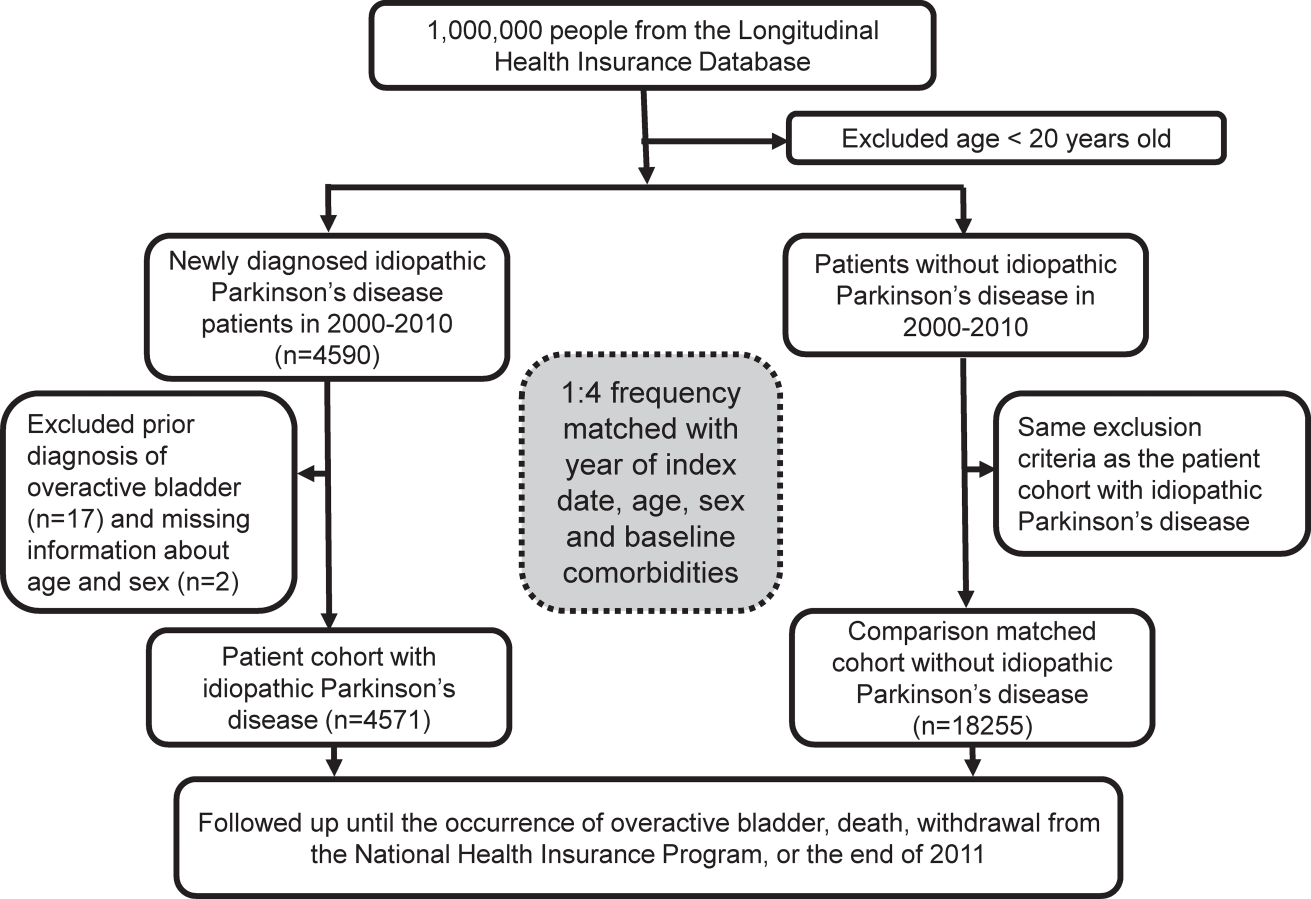
Flowchart presenting study subjects selection.

Patients aged older than 20 years who were newly diagnosed with IPD (ICD-9-CM code 332.0) between 2000 and 2010 were enrolled as the IPD cohort. The date of IPD diagnosis was defined as the index date. Controls without IPD (non-IPD) were also selected from the LHID. We excluded patients manifested as atypical or secondary parkinsonism (ICD-9-CM code 332.1). Patients in both cohorts who had been previously diagnosed with OAB (ICD-9-CM code 596.51) at outpatient clinics before the index date were excluded. For each patient with IPD, four non-IPD controls were matched for year of index date, age, sex, and similar proportions of baseline comorbidities, including benign prostate hyperplasia (ICD-9-CM code 600), stress incontinence (ICD-9-CM code 625.6), diabetes (ICD-9-CM code 250), and cerebrovascular diseases (ICD-9-CM codes 430–438). All patients included in the study were followed up between the index date and either the occurrence of OAB, death, withdrawal from the NHIP, or the end of the study period in 2011 (Fig 1).

We used clinical diagnoses of OAB according to the ICD-9-CM code of hypertonicity of bladder (ICD-9-CM code 596.51) to specify the correlation of detrusor overactivity and IPD. We did not choose the ICD codes for symptoms involving urinary system (ICD-9-CM code 788.X), such as retention of urine (ICD-9-CM code 788.2) or urinary incontinence (ICD-9-CM code 788.3), because they are non-specific urinary symptoms.

### Statistical analysis

Basic demographic data characteristics and comorbidities were compared between the two cohorts using t-test for age and chi-squared test for sex and comorbidities. The incidence rate of OAB (per 10,000 person-years) was calculated for both cohorts. The risk of developing OAB was estimated for patients with and without IPD, as well as those with associated risk factors. Hazard ratios (HRs) and 95% confidence intervals (CIs) were calculated by applying univariate and multivariate Cox regression models (assuming constant proportional hazards). The cumulative incidence of OAB in IPD and non-IPD cohorts were plotted using the Kaplan–Meier method, and their differences were examined using the log-rank test. Furthermore, the risk of developing OAB was evaluated and stratified by sex, age, and comorbidities in each cohort. All data analyses were performed using SAS statistical software (Version 9.4, SAS Institute, Cary, NC, United States), and the two-sided significance level was set at p < 0.05.

## Results

In total, 4,571 and 18,255 patients were included in the IPD and non-IPD cohorts, respectively, (Table 1), and distributions of age, sex, and comorbidities were similar between both groups. The mean (± standard deviation; SD) ages of IPD and non-IPD cohorts were 72.5 (± 10.7 years) and 71.8 (± 10.8 years), respectively. In each cohort, approximately 49.6% of the patients were women. The major comorbidities in the IPD and non-IPD cohorts were benign prostate hyperplasia (10.4% vs. 10.4%), stress incontinence (1.25% vs. 1.11%), diabetes (23.2% vs. 23.3%), and cerebrovascular disease (19.7% vs. 19.7%). The mean follow-up periods were 5.0 years for the IPD cohort and 5.3 years for the non-IPD cohort.

**Table 1:** Characteristics between patients with and without idiopathic Parkinson’s disease.

	Idiopathic Parkinson’s disease				p-value
	Yes		No		
	(N = 4,571)		(N = 18,255)		
	n	%	n	%	
Age, year					0.99
≤64	863	18.9	3449	18.9	
65–74	1587	34.7	6341	34.7	
75–84	1777	38.9	7099	38.9	
≥85	344	7.53	1366	7.48	
Mean (SD)	72.5	(10.7)	71.8	(10.8)	
Sex					0.99
Female	2267	49.6	9047	49.6	
Male	2304	50.4	9208	50.4	
Comorbidity					
Benign prostate hyperplasia	475	10.4	1892	10.4	0.99
Stress incontinence	57	1.25	203	1.11	0.44
Diabetes	1062	23.2	4246	23.3	0.97
Cerebrovascular diseases	900	19.7	3589	19.7	0.96

Abbreviation: SD, standard deviation.

The incidence rates of OAB were approximately 14.5 and 6.37 per 10,000 person-years for the IPD and the non-IPD cohorts, respectively (Table 2). The risk of OAB was significantly higher in the IPD cohort than in the non-IPD cohort (HR = 2.31, 95% CI = 1.51–3.52), and the risk of OAB was higher for patients with the comorbidities of benign prostate hyperplasia (HR = 2.88, 95% CI = 1.63–5.07) and stress incontinence (HR = 3.19, 95% CI = 1.01–10.1). It is noteworthy that the risk of OAB remained higher in the IPD cohort than in the non-IPD cohort (multivariate-adjusted HR = 2.30, 95% CI = 1.51–3.51) after adjustment for these comorbidities.

**Table 2:** Incidence rates and hazard ratios (HR) of overactive bladder for idiopathic Parkinson’s disease and potential risk factors in multivariate cox regression model analysis.

Variables	Event	PY	Rate^#^	HR (95%CI)	
				Univariate	Multivariate^&^
**Idiopathic Parkinson’s disease**					
**No**	62	97328	6.37	1.00	1.00
**Yes**	33	22717	14.5	2.31(1.51, 3.52)***	2.30(1.51, 3.51)***
**Age, year**					
**≤64**	17	26886	6.32	1.00	1.00
**65–74**	42	46106	9.11	1.45(0.82, 2.54)	-
**75–84**	32	41269	7.75	1.27(0.70, 2.29)	-
**≥85**	4	5784	6.92	1.15(0.39, 3.44)	-
**Sex**					
**Male**	39	59371	6.57	1.00	1.00
**Female**	56	60674	9.23	1.40(0.93, 2.11)	-
**Comorbidity**					
**Benign prostate hyperplasia**					
**No**	80	112018	7.14	1.00	1.00
**Yes**	15	8027	18.7	2.88(1.63, 5.07)***	2.94(1.66, 5.18)***
**Stress incontinence**					
**No**	92	118816	7.74	1.00	1.00
**Yes**	3	1229	24.4	3.19(1.01, 10.1)*	3.44(1.09, 10.9)*
**Diabetes**					
**No**	69	94529	7.30	1.00	1.00
**Yes**	26	25517	10.2	1.42(0.90, 2.23)	-
**Cerebrovascular diseases**					
**No**	82	100286	8.18	1.00	1.00
**Yes**	13	19760	6.58	0.82(0.46, 1.48)	-

CI: confidence interval.

Rate^#^: incidence rate, per 10,000 person-years.

^&^: multivariate analysis including comorbidities of benign prostate hyperplasia, and stress incontinence.

*p<0.05.

**p<0.01.

***p<0.001.

**Fig 2:**
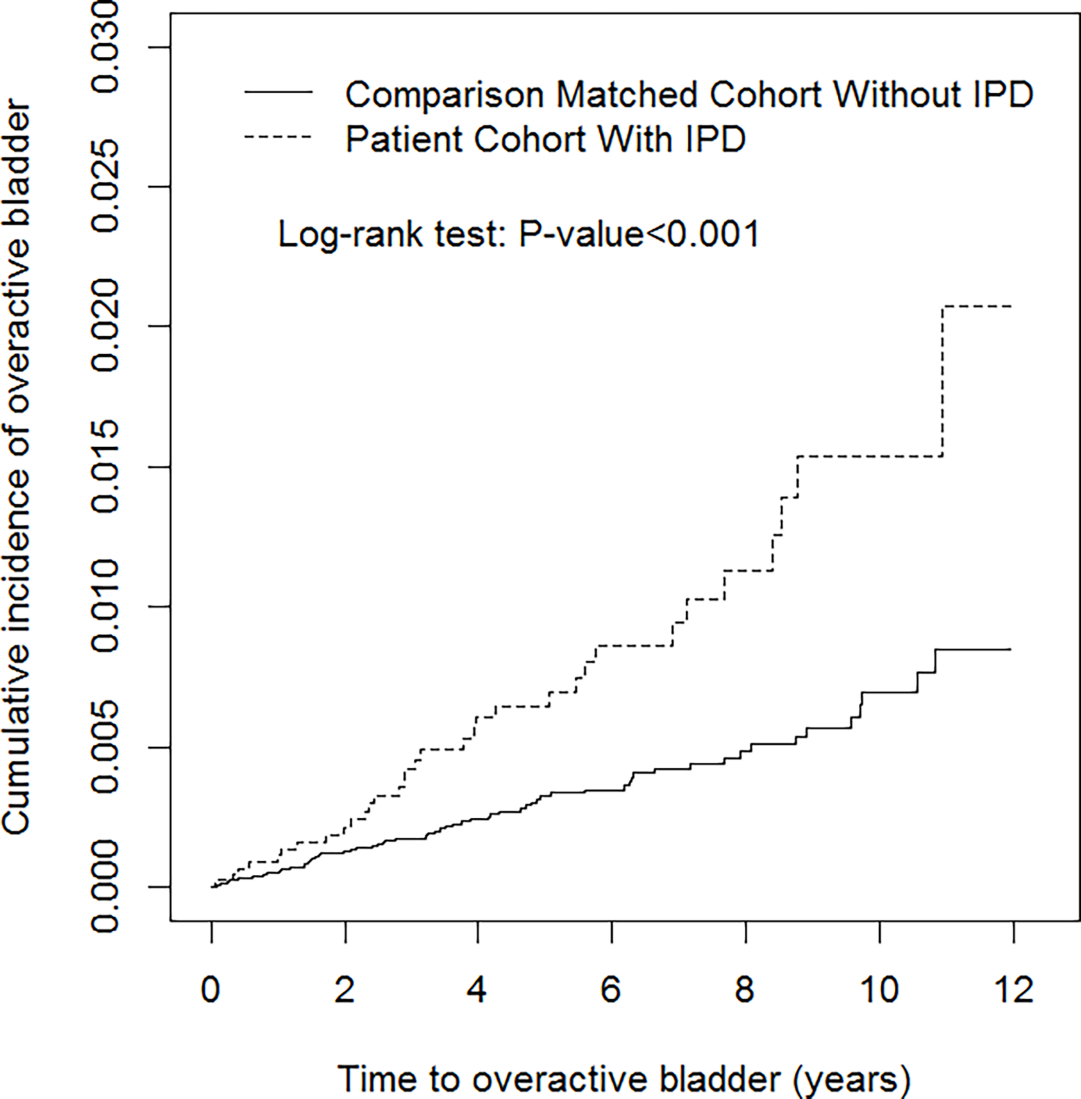
Cumulative incidence of overactive bladder (OAB) for patients with and without idiopathic Parkinson’s disease (IPD).

The cumulative incidence of OAB estimated using the Kaplan–Meier analysis was significantly higher in the IPD cohort than that in the non-IPD cohort by the end of the 12-year follow-up (Fig 2, log-rank test, p < 0.001). Our population-based cohort study revealed that the cumulative incidence of OAB was 0.65% at the fifth year and 1.54% at the tenth year after IPD diagnosis.

We further estimated the risk of OAB in the IPD and the non-IPD cohorts stratified by sex, age, and comorbidity status (Table 3). A positive association between IPD and OAB was observed in patients of both sexes (female patients: multivariate-adjusted HR = 2.47, 95% CI = 1.29–4.76; male patients: multivariate-adjusted HR = 2.19, 95% CI = 1.26–3.81), patients without comorbidities (multivariate-adjusted HR = 2.44, 95% CI = 1.35–4.41), and patients with comorbidities (multivariate-adjusted HR = 2.18, 95% CI = 1.19–3.98). For the age-specific IPD to non-IPD cohort HR, the risk of OAB was increased among patients aged 65–74 years (multivariate-adjusted HR = 3.65, 95% CI = 1.99–6.71).

**Table 3:** Subgroup analysis for incidence rates and hazard ratios (HR) of overactive bladder between patients with and without idiopathic Parkinson’s disease, stratified by sex, age, and comorbidity status.

Characteristics	Idiopathic Parkinson’s disease						HR (95% CI)	
	Yes			No			Univariate	Multivariate^&^
	Event	PY	Rate^#^	Event	PY	Rate^#^		
**Sex**								
**Female**	19	11597	16.4	37	49078	7.54	2.19(1.26, 3.81)**	2.19(1.26, 3.81)**
**Male**	14	11120	12.6	25	48250	5.18	2.47(1.28, 4.76)**	2.47(1.29, 4.76)**
**Age, year**								
**≤64**	5	5151	9.71	12	21735	5.52	1.77(0.62, 5.03)	1.79(0.63, 5.08)
**65–74**	19	8730	21.8	23	37375	6.15	3.63(1.98, 6.67)***	3.65(1.99, 6.71)***
**75–84**	9	7713	11.7	23	33556	6.85	1.71(0.79, 3.70)	1.72(0.79, 3.72)
**≥85**	0	1123	0.00	4	4662	8.58	-	-
**Comorbidity**^§^								
**No**	17	14139	12.0	31	61179	5.07	2.44(1.35, 4.41)**	2.44(1.35, 4.41)**
**Yes**	16	8578	18.7	31	36149	8.58	2.18(1.19, 3.98)*	2.18(1.19, 3.98)*

CI: confidence interval.

Rate^#^: incidence rate, per 10,000 person-years.

^&^: multivariate analysis including comorbidities of benign prostate hyperplasia, and stress incontinence.

^§^: subjects with any comorbidity of benign prostate hyperplasia, stress incontinence, diabetes, or cerebrovascular diseases were classified into the comorbidity group.

*p<0.05.

**p<0.01.

***p<0.001.

## Discussion

IPD is the most common form of parkinsonism. Although there are other possible causes of parkinsonism, including Parkinson-plus syndromes, such as multiple system atrophy (MSA), or secondary parkinsonism, such as toxin-related diseases, atherosclerosis, and metabolic diseases, clinical phenomena may be similar in the initial disease stages, despite the diverse etiologies of pathogenesis. However, the pathophysiology of MSA differs from that of IPD; for example, urinary disturbances are more prominent in patients with MSA [4–6,12]. Some authors have argued that the high prevalence of urinary symptoms determined in earlier studies may have resulted from a possible misclassification of disease entities, such as the inclusion of some patients with atypical parkinsonism, particularly MSA [12,13]. We excluded the patients manifested as atypical or secondary parkinsonism (ICD-9-CM code 332.1) to avoid such possible misclassification bias.

The prevalence of LUTS in cross-sectional studies of PD ranges from 27% to 85%. Nocturia is the most commonly reported symptom (57%–86%), followed by frequency (32%–71%) and urgency (32%–68%) [6]. From literature review, detrusor overactivity is the commonest cystometric abnormality in patients with IPD. The current proposed hypothesis is that basal ganglia output has an overall inhibitory effect on the micturition reflex in healthy individuals. The basal ganglia “normally” inhibits the micturition reflex via D1 receptors. In patients with IPD, degeneration of the dopaminergic neurons in the substantia nigra and a subsequent loss of striatial dopamine concentrations may lead to exaggerated micturition reflex through an inability to activate D1-mediated tonic inhibition [5,12–15]. Detrusor overactivity is considered the consequence of exaggerated micturition reflex. In brief, an altered frontal cortex-basal ganglia circuit may cause detrusor overactivity in patients with IPD [13–15]. We would like to specify the correlation of detrusor overactivity and IPD. Therefore, we did not choose the ICD codes for symptoms involving urinary system (ICD-9-CM code 788.X), such as retention of urine (ICD-9-CM code 788.2) or urinary incontinence (ICD-9-CM code 788.3), which are non-specific urinary symptoms. We chose the ICD code (ICD-9-CM code 596.51: hypertonicity of bladder) which was supposed to be specific for detrusor overactivity. Our long-term follow-up study used an adequate-sized population, and results showed a 2.3-fold increased risk of OAB among patients with IPD after considering comorbidities such as benign prostate hyperplasia and stress incontinence.

Information regarding the disease course of urinary symptoms appearance in patients with IPD is extremely limited [6]. For example, Bonnet et al. revealed that urinary disturbances were believed to occur 6 years after the onset of motor symptoms in patients with IPD. The main urinary symptom was urgency to void, and the urodynamic analysis demonstrated detrusor hyperreflexia and normal urethral sphincter function [16]. Besides, the results obtained by Wenning et al. corroborate our findings, because they reported a median latency of 12 years from IPD diagnosis to the symptom of urinary incontinence in a small-scale postmortem confirmed IPD patients [17]. We observed that the incidence rate of newly diagnosed OAB in the IPD cohort was 14.5 per 10,000 person-years. Our cohort study also indicated that the cumulative incidence of OAB, which could be considered as long-term probability, was 0.65% at the fifth year and 1.54% at the tenth year after IPD diagnosis. For the IPD patients in our cohort who were newly diagnosed with OAB during the study period, the mean interval between diagnosis of IPD and OAB occurrence was approximately 5 years.

We also evaluated potential “second hit” factors relating to comorbidities that may also play a role in OAB. For example, benign prostate hyperplasia is a common coexisting disorder in older men with IPD. Studies have shown that male patients with bladder outlet obstruction complain of difficulties in voiding, such as hesitancy and poor flow, and may also experience urgency because the obstruction itself can cause detrusor overactivity [5,12]. In addition, the prevalence of stress incontinence is high for older women. Moreover, it has been determined that idiopathic detrusor overactivity may occur, which could be a result of previous cerebral ischemia in asymptomatic individuals [6]. In this study, we found that benign prostate hyperplasia and stress incontinence were significantly associated with an increased risk of OAB. However, we also found that the risk of OAB remained high in patients with IPD after adjustment for the effects of benign prostate hyperplasia and stress incontinence. Specifically, after considering sex-specific common cofounders, our results show that it is highly probably that IPD is an independent medical condition that causes OAB symptoms.

Gray et al. found that lower urinary tract dysfunction in PD is related to age but not to the disease itself [18], and Campos-Sousa et al. demonstrated that only the age of patients was correlated with urinary dysfunctions [19]. In our study, age alone did not significantly increase the incidence of OAB. However, the most significant association between IPD and OAB was observed among patients aged 65–74 years. This suggests that IPD may be an independent risk factor for OAB in this age group, which could impact the comprehensive care of urinary symptoms in patients with IPD through effective IPD therapy. However, because the severity of IPD cannot be determined through reimbursement-based health databases, this hypothesis could not be validated. Therefore, we recommend that additional studies are conducted to verify the age-related elevated risk of OAB among patients with IPD.

This study had several limitations that should be considered. First, ICD-9-CM codes were used, because the original purpose was to apply reimbursement to identify cohorts at the NHIRD. We also assumed that a proficient disease diagnosis had been made based on an overall clinical assessment, laboratory data, neuroimages, and related studies. In summary, although all insurance claims in the NHIRD were scrutinized by medical doctors in clinical practice and then peer-reviewed by board-specific specialists according to the standard diagnosed criteria, it could be considered that the ICD-9-CM codes used in the current study may not have the same quality of operative definitions as those of a prospective well-designed clinical study. Secondly, this study does not include information relating to IPD severity, as we were prohibited from viewing the individual charts of participants in accordance with the study principles of the NHIRD. Consequentially, it was not possible to precisely correlate the natural history of IPD with the development of OAB. The stage of IPD and motor function in patients with IPD might be contributing factors to the OAB symptoms. In our study, we did not have the information regarding the disease stage or motor function status in patients with IPD. Further study could be conducted to verify this issue. Thirdly, evidence derived from a retrospective cohort study is usually of lower quality than that derived from a prospective type, because of sources of inherent bias (such as selection bias). For example, the use of codes in this study may result in non-differential (or random) misclassification, which may bias results toward the null [20]. In fact, it is possible that our findings tend to underestimate the risk of OAB occurrence among IPD patients, if non-differential misclassification exists. Finally, depression is considered an important non-motor symptom in patients with IPD. The symptoms of OAB may cause psychosocial distress in patients. We emphasize the correlation of IPD and OAB according to the pathophysiological model of dopamine depletion resulting in exaggerated micturition reflex. The comorbidities of depression and related mental illness were not controlled in this study. Further study could be conducted to explore the relationship of depression and IPD, and occurrence of OAB.

In conclusion, the results of this nationwide population-based cohort study showed that the risk of OAB was significantly higher in patients with IPD compared with the matched non-IPD patients. In addition, the long-term probability of occurrence of OAB in the IPD cohort at tenth year was 1.54%. For the IPD patients in our cohort who were diagnosed with OAB during the study period, the mean interval between the diagnosis of IPD and the occurrence of OAB was approximately 5 years. Therefore, additional studies are required to investigate the causal relationship and identify risk factors between IPD and OAB.
